# Supplementary material for: Mapping the immune environment in clear cell renal carcinoma by single-cell genomics
Source: Commun Biol. 2021 Jan 27;4:122. doi: 10.1038/s42003-020-01625-6 (PMC7840906; doi:10.1038/s42003-020-01625-6)
Supplement: Supplementary file 2 — Supplementary Information [file 42003_2020_1625_MOESM2_ESM.pdf]

Title: Mapping the Immune Environment in Clear Cell Renal Carcinoma by Single-Cell Genomics

Authors: Nick Bocherding[1,2]†, Ajaykumar Vishwakarma[3,4,7]†, Andrew P. Voigt[2], Andrew Bellizzi[5], Jacob Kaplan[5], Kenneth Nepple[6], Aliasger K. Salem[3], Russell W. Jenkins[4, 7 *], Yousef Zakharia[6, 8*], Weizhou Zhang[9 *]

Affiliations:
[1] Department of Pathology and Immunology, Washington University School of Medicine, St Louis, MO
[2] Medical Science Training Program, University of Iowa, Iowa City, IA
[3] Department of Pharmaceutical Sciences and Experimental Therapeutics, College of Pharmacy, University of Iowa, Iowa City, IA
[4] Department of Medicine, Massachusetts General Hospital Cancer Center, Harvard Medical School, Boston, MA
[5] Department of Pathology, University of Iowa Hospitals and Clinics, Iowa City, IA
[6] Department of Urology, University of Iowa Hospitals and Clinics, Iowa City, IA
[7] Laboratory of Systems Pharmacology, Harvard Program in Therapeutic Science, Harvard Medical School, Boston, MA
[8] Department of Internal Medicine, University of Iowa Hospitals and Clinics, Iowa City, IA
[9] Department of Pathology, Immunology, and Laboratory Medicine, University of Florida, Gainesville, FL

† Authors contributed equally to this work

* Correspondence:

rjenkins@mgh.harvard.edu (R.W.J)

yousef-zakharia@uiowa.edu (Y.Z)

zhangw@ufl.edu (W.Z)

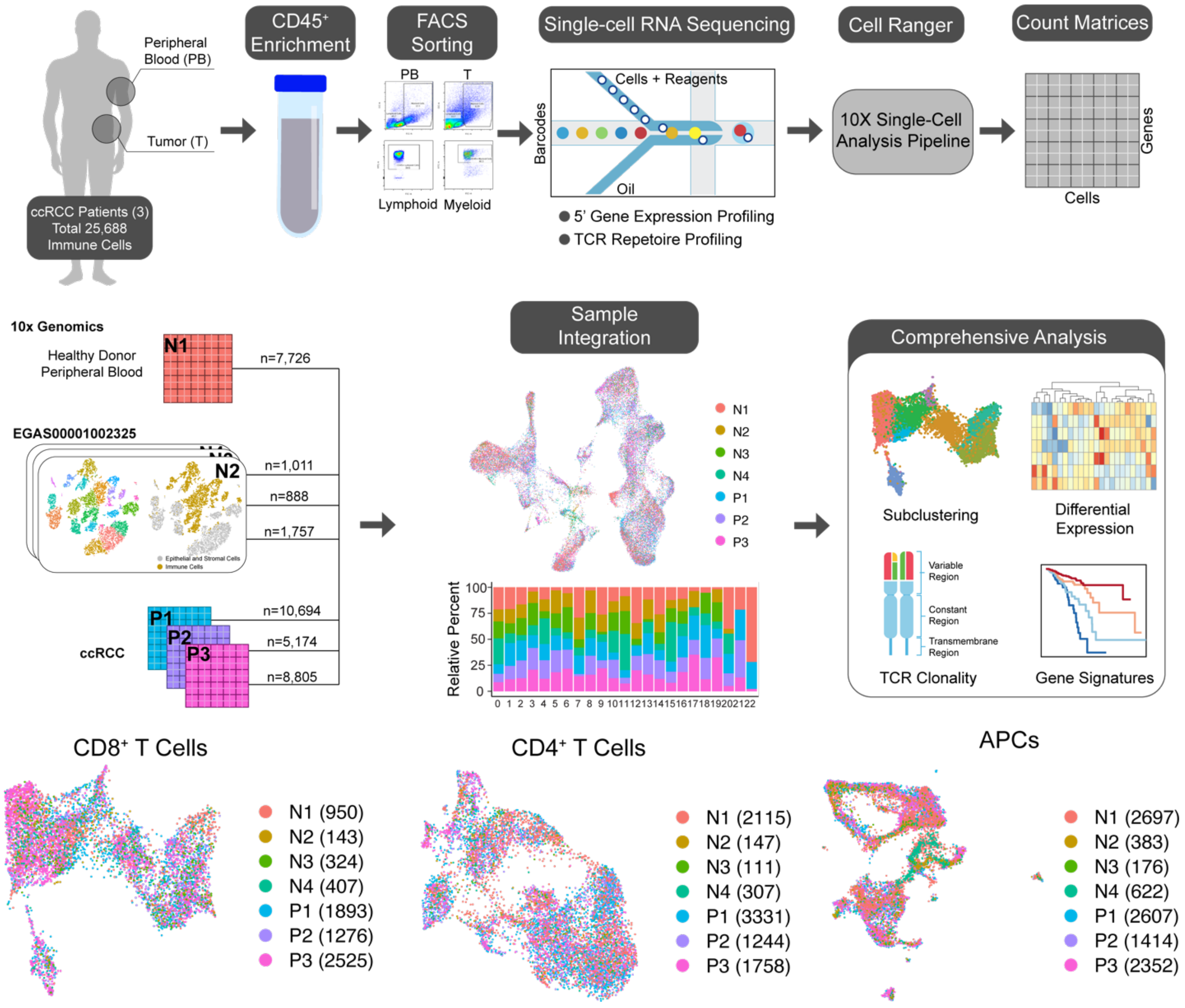

Supplementary Figure 1: Workflow for SCRS of isolated lymphoid and myeloid immune cell populations from ccRCC subjects and computational analysis. SCRS data across ccRCC (both tumor and paired peripheral blood) and data derived from 10x Genomics and EGAS00001002325 were integrated using SCTtransform approach to form a UMAP of 37,055 from 7 different samples and acting as a basis for subclustering and further analysis by cell type. During the subclustering analysis of CD8^{+}, CD4^{+} T cells, and APCs, single-cells were re-integrated by patient sample, patients and corresponding numbers of cells indicated.

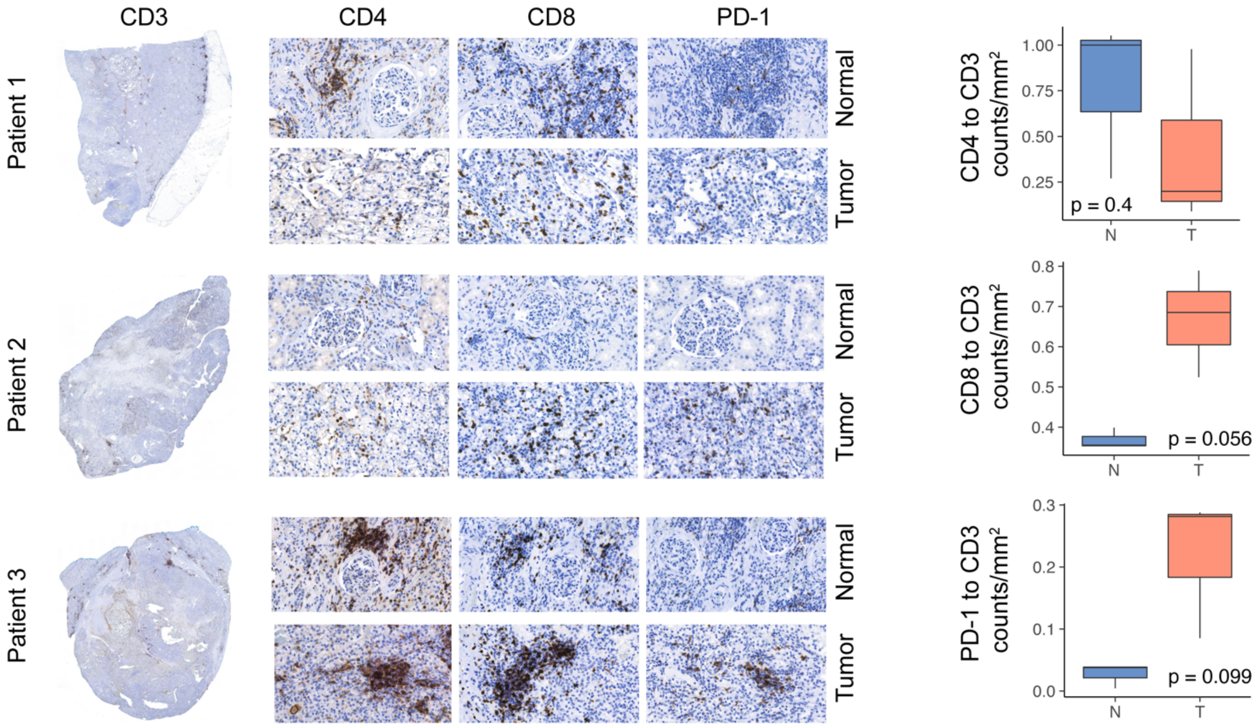

Supplementary Figure 2: Immunohistochemical results for T cell staining in paired normal and tumor tissue with quantified ratios of counts per unit area.

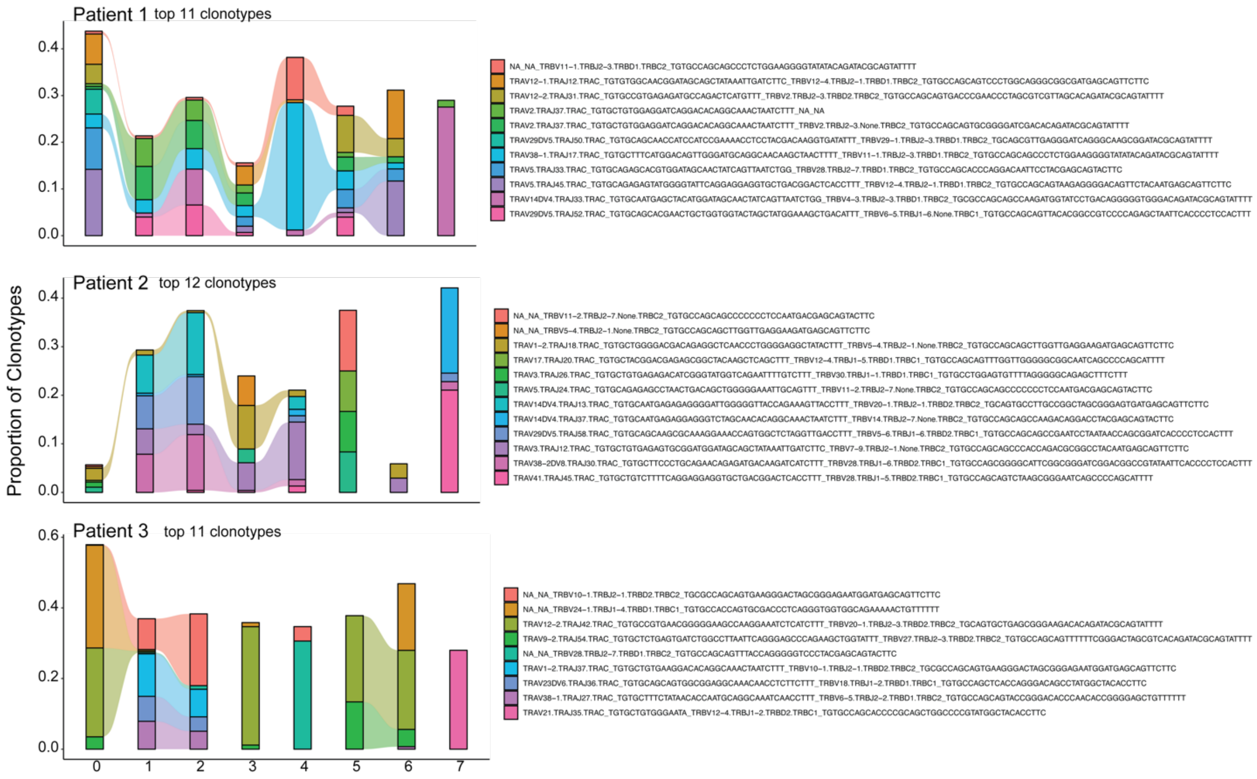

Supplementary Figure 3: Top clonotypes for CD8$^+$ T cells for each patient by proportion in each subcluster.

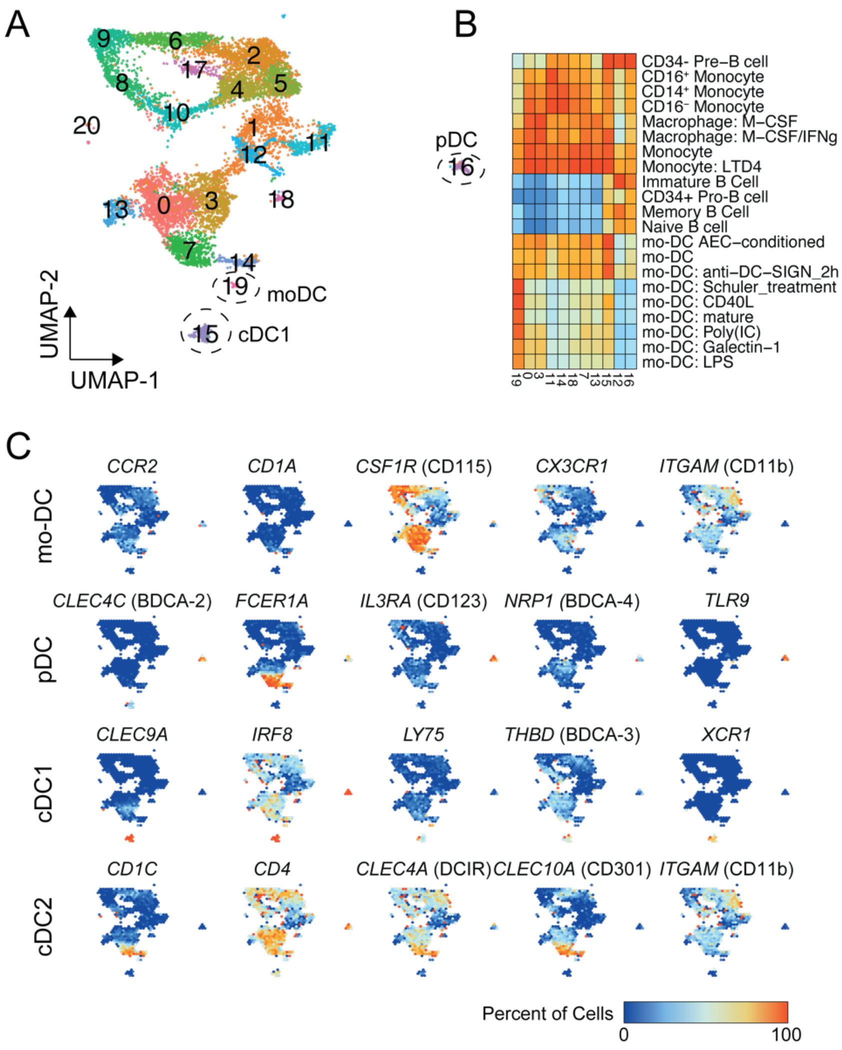

Supplementary Figure 4: A. UMAP subclustering of myeloid cells (original clusters 4, 6, 10, 13, 15, and 20). **B**. Normalized correlation values for predicted immune cell phenotypes based on the SingleR R package for selected subclusters. **C**. Percent of cells expressing canonical immune cell markers across the UMAP.

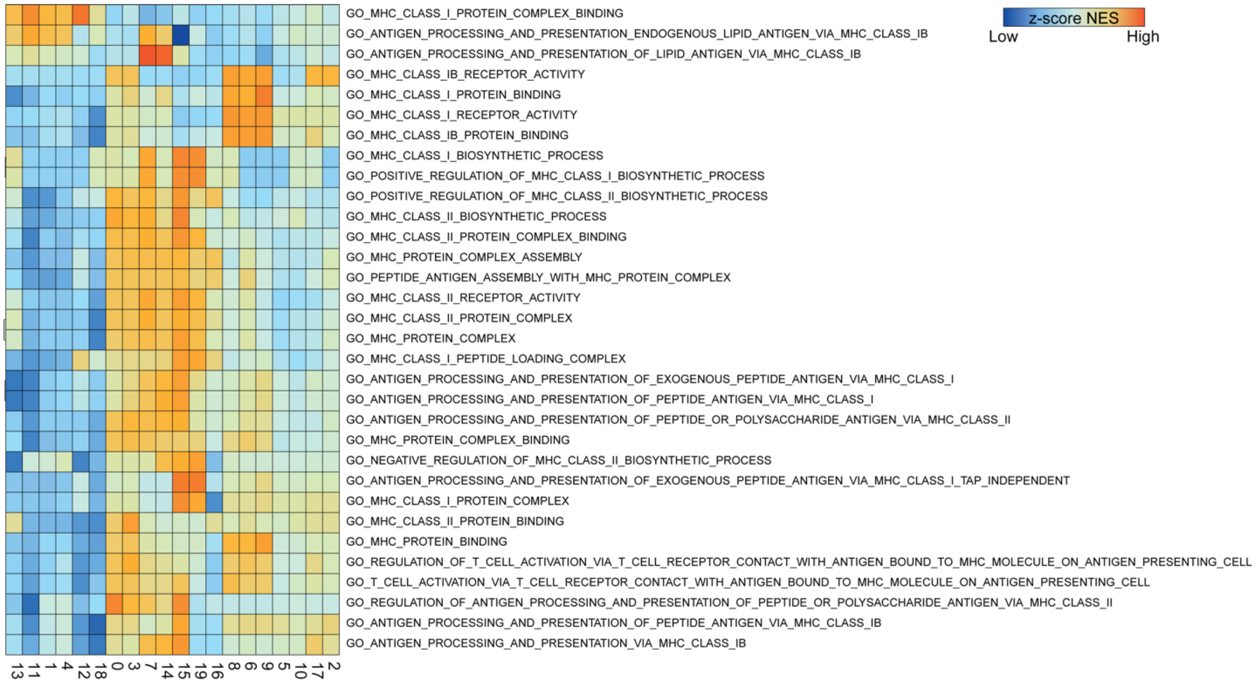

Supplementary Figure 5: Z-transformed normalized enrichment scores from ssGSEA for MHC-related gene sets in the C5 library of the Molecular Signature Database by APC/Myeloid subcluster.

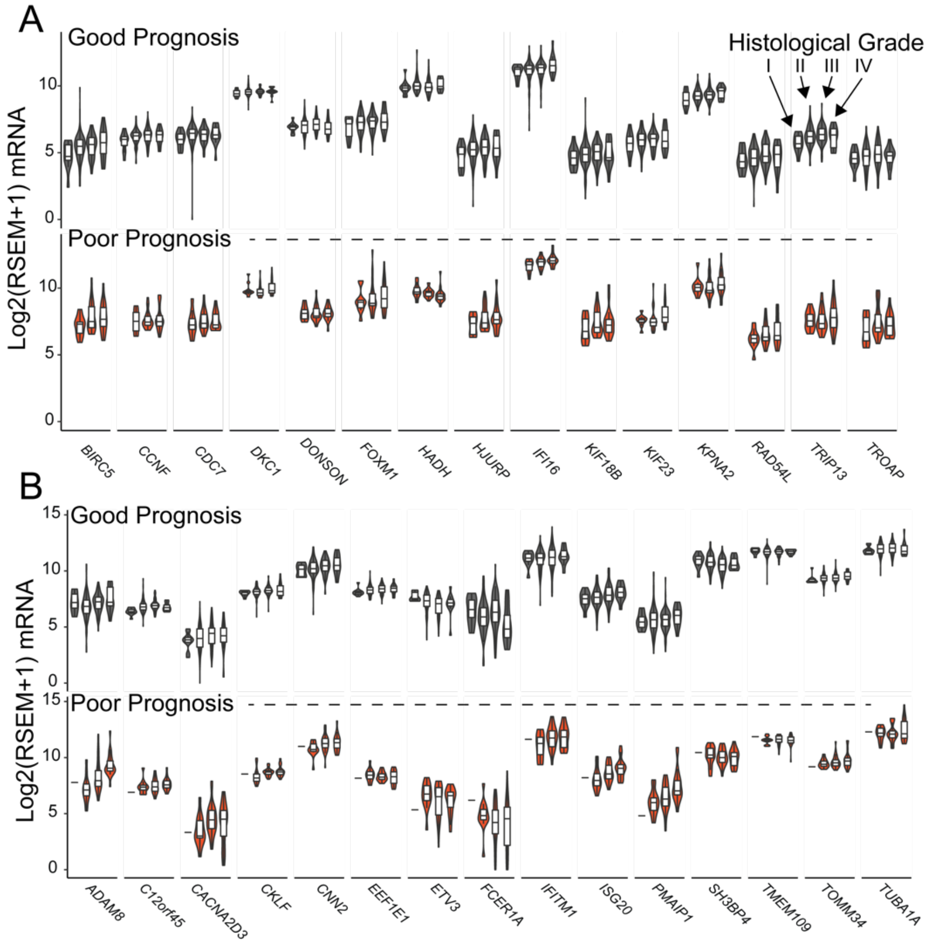

Supplementary Figure 6: A. mRNA expression of signature genes for CD8_6 (upper panel) and TAM_3 (lower panel) by histological grade (x-axis) and k-nearest neighbor model prediction.

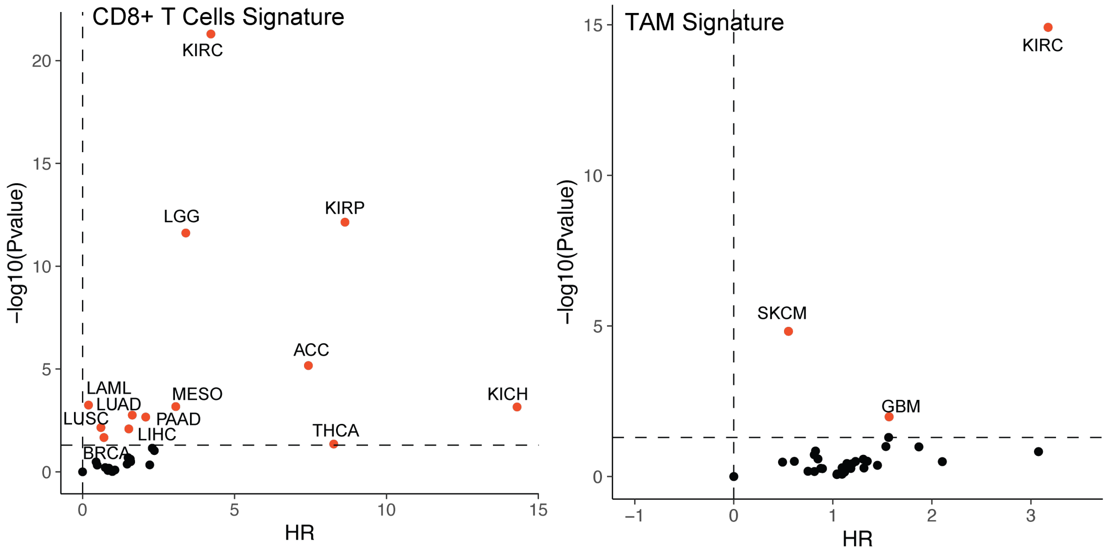

Supplementary Figure 7. Cox hazard ratio versus logrank -log10(p-value) for sig CD8_6 (right panel) and TAM_3 (left panel) applied across 33 TCGA data set. Points in red are significant and labeled with the corresponding cancer abbreviations.

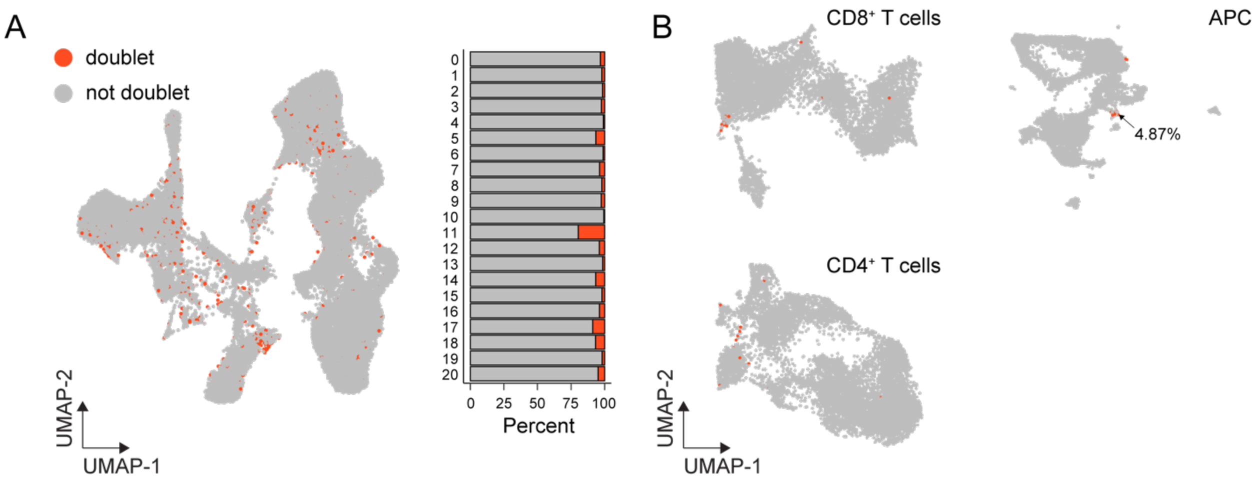

Supplementary Figure 8: A. UMAP plot and corresponding percent bar graph by cluster for doublet estimation using scDblFinder R package across all cells in the integrated data set. Doublets defined as density estimated with log2(x+1) ≥ 3. **B**. Doublet assignments for individual cells types. All new subclusters had < 2% of doublet assignments, except APC subcluster 12 (arrow) with 4.87% of the cluster assigned as doublet/multiplet.

Supplementary Methods

Highthroughput IHC quantification

Normal and ccRCC tumor samples from the 3 patients were formalin-fixed and paraffin-embedded samples. After antigen-retrieval, samples were stained with indicated anitbodies . Digital images of 24 IHC-stained slides were obtained using the 3DHistech P1000 Panoramic Scanner (3DHistech, Budapest, Hungary). Each slide was annotated in CaseViewer (3DHistech) for tumor and or normal tissue. Areas with abundant artifact were annotated and subtracted. Image-based algorithms were created for detecting positive cells based on stain intensity using QuantCenter Image Analysis Framework software (3DHistech) and normalized per area analyzed.
