# Supplementary material for: Mapping the immune environment in clear cell renal carcinoma by single-cell genomics
Source: Commun Biol. 2021 Jan 27;4:122. doi: 10.1038/s42003-020-01625-6 (PMC7840906; doi:10.1038/s42003-020-01625-6)
Supplement: Supplementary file 3 — Description of Additional Supplementary Files [file 42003_2020_1625_MOESM3_ESM.pdf]

Description of Additional Supplementary Files

File Name: Supplementary Data 1
Description: VDJ clonotype assignments by single-cell barcodes

File Name: Supplementary Data 2
Description: 2 Differential genes comparing peripheral-blood and tumorinfiltrated CD4+ T cells

File Name: Supplementary Data 3
Description: Summary of mean and median features across integrated single-cell samples.

File Name: Supplementary Data 4
Description: Dimensional reduction inputs for CD8+ T Cells, CD4+ T cell and Myeloid cell analyses.

File Name: Supplementary Data 5
Description: Lineage-specific gene lists for cell type assignment.
