# Supplementary material for: Mapping the immune environment in clear cell renal carcinoma by single-cell genomics
Source: Commun Biol. 2021 Jan 27;4:122. doi: 10.1038/s42003-020-01625-6 (PMC7840906; doi:10.1038/s42003-020-01625-6)
Supplement: Supplementary file 9 — Reporting Summary [file 42003_2020_1625_MOESM9_ESM.pdf]

nature research

Corresponding author(s): Russell W. Jenkins
Yousef Zakharia
Weizhou Zhang

Last updated by author(s): 11/27/2020

Reporting Summary

Nature Research wishes to improve the reproducibility of the work that we publish. This form provides structure for consistency and transparency in reporting. For further information on Nature Research policies, see Authors & Referees and the Editorial Policy Checklist.

Statistics

For all statistical analyses, confirm that the following items are present in the figure legend, table legend, main text, or Methods section.

n/a	Confirmed	
☐	☒	The exact sample size (*n*) for each experimental group/condition, given as a discrete number and unit of measurement
☐	☒	A statement on whether measurements were taken from distinct samples or whether the same sample was measured repeatedly
☐	☒	The statistical test(s) used AND whether they are one- or two-sided<br>*Only common tests should be described solely by name; describe more complex techniques in the Methods section.*
☒	☐	A description of all covariates tested
☐	☒	A description of any assumptions or corrections, such as tests of normality and adjustment for multiple comparisons
☐	☒	A full description of the statistical parameters including central tendency (e.g. means) or other basic estimates (e.g. regression coefficient) AND variation (e.g. standard deviation) or associated estimates of uncertainty (e.g. confidence intervals)
☐	☒	For null hypothesis testing, the test statistic (e.g. *F*, *t*, *r*) with confidence intervals, effect sizes, degrees of freedom and *P* value noted<br>*Give P values as exact values whenever suitable.*
☒	☐	For Bayesian analysis, information on the choice of priors and Markov chain Monte Carlo settings
☐	☒	For hierarchical and complex designs, identification of the appropriate level for tests and full reporting of outcomes
☒	☐	Estimates of effect sizes (e.g. Cohen's *d*, Pearson's *r*), indicating how they were calculated

Our web collection on statistics for biologists contains articles on many of the points above.

Software and code

Policy information about availability of computer code

Data collection

Single-cell library preparation was carried out as per the 10X Genomics Chromium Single Cell 5' Library and Gel Bead Kit v2 #1000014 (10x Genomics, Pleasanton, CA). Cell suspensions were loaded onto a Chromium Single-Cell Chip along with the reverse transcription (RT) master mix and single cell 5'gel beads, aiming for 7,500 cells per channel. Following generation of single-cell gel bead-in-emulsions (GEMs), reverse transcription was performed using a C1000 Touch Thermal Cycler (Bio-Rad Laboratories, Hercules, CA); 13 cycles were used for cDNA amplification. Amplified cDNA was purified using SPRIselect beads (Beckman Coulter, Lane Cove, NSW, Australia) as per the manufacturer's recommended parameters. Post-cDNA amplification reaction QC and quantification was performed on the Agilent 2100 Bioanalyzer using the DNA High Sensitivity chip. For input into the gene expression library construction, 50ng cDNA and 14 cycles was used. To obtain TCR repertoire profile, VDJ enrichment was carried out as per the Chromium Single Cell V(D)J Enrichment Kit, Human T Cell #1000005 (10x Genomics) using the same input samples. Sequencing libraries were generated with unique sample indices for each sample and quantified. Libraries were sequenced on an Illumina HiSeq 4000 using a 150-pair-end sequencing kit. Gene expression FASTQ files were aligned to the human genome (GRCh38) using the CellRanger v2.2 pipeline, while clonotype sequencing was aligned to the vdj_GRCh38_alts_ensembl genome build provided by the manufacturer.
T cell receptor sequences for single-cell mRNA sequencing are available at the Gene Expression Omnibus (GEO) at GSE121638.

Data analysis

Initial processing of cells isolated from ccRCC patients; Patient 1 (n=10,694), Patient 2 (n=5,174) and Patient 3 (n=9,805) were processed and integrated with the above samples using the Seurat R package (v3.0.2). We removed cells with percentage of mitochondrial genes > 15% and UMI > 5,000 to control for multiplets. Samples were normalized using the SCTtransform approach with default settings. Preparation for integration used 3,000 anchor features and PrepSCTIntegration. The integration of sequencing runs occurred with the SCT-transformed data. The dimensional reduction to form the UMAP utilized the top 30 calculated dimensions and a resolution of 0.7. The processed data and code for all analyses is available at https://github.com/ncborcherding/ccRCC.

For manuscripts utilizing custom algorithms or software that are central to the research but not yet described in published literature, software must be made available to editors/reviewers. We strongly encourage code deposition in a community repository (e.g. GitHub). See the Nature Research guidelines for submitting code & software for further information.

Data

Policy information about availability of data

All manuscripts must include a data availability statement. This statement should provide the following information, where applicable:

- Accession codes, unique identifiers, or web links for publicly available datasets
- A list of figures that have associated raw data
- A description of any restrictions on data availability

Gene Expression Omnibus (GEO) at GSE121638.

Field-specific reporting

Please select the one below that is the best fit for your research. If you are not sure, read the appropriate sections before making your selection.

☒ Life sciences ☐ Behavioural & social sciences ☐ Ecological, evolutionary & environmental sciences

For a reference copy of the document with all sections, see nature.com/documents/nr-reporting-summary-flat.pdf

Life sciences study design

All studies must disclose on these points even when the disclosure is negative.

Sample size	n=3 (paired primary human tumor and blood from ccRCC) Addition of 4 controls.
Data exclusions	No data were excluded
Replication	n/a
Randomization	n/a
Blinding	n/a

Reporting for specific materials, systems and methods

We require information from authors about some types of materials, experimental systems and methods used in many studies. Here, indicate whether each material, system or method listed is relevant to your study. If you are not sure if a list item applies to your research, read the appropriate section before selecting a response.

Materials & experimental systems

n/a	Involved in the study	
☐	☒	Antibodies
☒	☐	Eukaryotic cell lines
☒	☐	Palaeontology
☒	☐	Animals and other organisms
☐	☒	Human research participants
☒	☐	Clinical data

Methods

n/a	Involved in the study	
☒	☐	ChIP-seq
☐	☒	Flow cytometry
☒	☐	MRI-based neuroimaging

Antibodies

Antibodies used	FITC anti-human CD45 antibody (BioLegend)
Validation	https://www.biolegend.com/en-us/products/fitc-anti-human-cd45-antibody-707

Human research participants

Policy information about studies involving human research participants

Population characteristics	The patients ranged from 67 to 74 years old; the primary clear cell renal cell carcinoma (ccRCC) tumor samples were of diverse tumor stages paired with fresh blood and sourced from male subjects. Tumor grades were histologically determined by a pathologist. Three ccRCC tumor specimens paired with individual blood samples were used in the study.
Recruitment	Three tumor samples of diverse tumor stages were selected

Ethics oversight | University of Iowa ethics board committee

Note that full information on the approval of the study protocol must also be provided in the manuscript.

Flow Cytometry

Plots

Confirm that:

☒ The axis labels state the marker and fluorochrome used (e.g. CD4-FITC).

☒ The axis scales are clearly visible. Include numbers along axes only for bottom left plot of group (a 'group' is an analysis of identical markers).

☒ All plots are contour plots with outliers or pseudocolor plots.

☒ A numerical value for number of cells or percentage (with statistics) is provided.

Methodology

Sample preparation	(CD45+ Hoechst-) single cell<br>suspensions generated from three ccRCC tumor samples and blood
Instrument	FACS ARIA sorter (BD Biosciences)
Software	FlowJo
Cell population abundance	Determined by single cell RNA sequencing (PTPRC+)
Gating strategy	Lymphoid and Myeloid gating (Supplementary Figure 1)

☒ Tick this box to confirm that a figure exemplifying the gating strategy is provided in the Supplementary Information.
